# History in Perspective: The prime pathological players and role of phytochemicals in Alzheimer’s disease

**DOI:** 10.1016/j.ibneur.2022.04.009

**Published:** 2022-04-28

**Authors:** Mohd Sajad, Rajesh Kumar, Sonu Chand Thakur

**Affiliations:** a Centre for Interdisciplinary Research in Basic Sciences, Jamia Millia Islamia, New Delhi 110025, India; b Department of Reproductive Bio-medicine, The National Institute of Health and Family Welfare, Baba Gang Nath Marg, Munirka, New Delhi 110067, India

**Keywords:** Alzheimer's Disease, Tau protein, β-amyloids, Neuroinflammation, Phytochemicals

## Abstract

Alzheimer's disease is a steadily progressive, irreversible neurological disorder that is most frequently categorized under the umbrella term "neurodegeneration". Several attempts are underway to clarify the pathogenic mechanisms, identify the aetiologies, and determine a pathway by which the therapeutic steps can be implemented. Oxidative stress is one of the pathogenic processes, which is commonly believed to be associated with neurodegenerative diseases. Accumulation of extracellular amyloid-β protein (Aβ), hyperphosphorylation of tau, initiation of neurometabolic reactions characterized by the loss of neuronal function and synaptic failure, and decreased or lost learning capability and memory function are the most central neuropathological characteristics of AD. According to the amyloid cascade hypothesis, the enhanced deposition of Aβ deposits and neurofibrillary tangles due to hyperphosphorylation of Tau activates the cascade reactions in the brain. These reactions affect the synaptic activity and activation of microglia, which results in neuroinflammation due to enhanced immune function. Plant-based phytochemicals have also been used long ago against several diseases. Phytoconstituents play a significant neuroprotective property by preventing the pathophysiology of the disease. In this review, we have discussed the formation and crosstalk between amyloid and tau pathologies as well as the effect of neuroinflammation on the progression of AD. We have specifically focused on the formation of NFT, β-amyloids, inflammation, and pathophysiology of AD and the role of phytochemicals in the prevention of AD.

## Highlights

• AD is an insidious, slowly progressive, and neurodegenerative disorder.
• Common symptoms are memory loss, difficulty in recalling, and understanding.
• β-amyloids and Neurofibrillary tangles are the main factors in AD pathogenesis.
• Activated microglia and oxidative stress have different effects on AD progression.
• Phytochemicals show a key role against AD by inhibiting several pathways.

## Graphical Abstract

**Figure:**
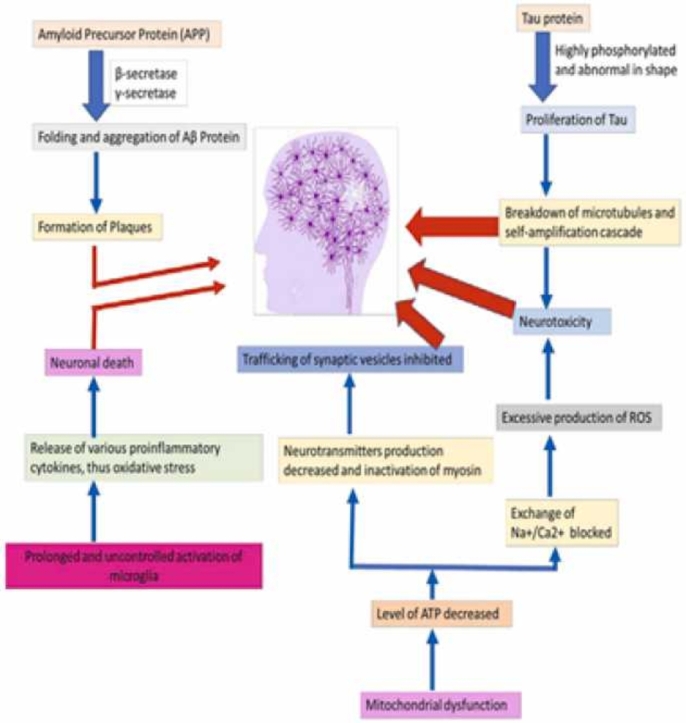

## 1 Introduction

**Fig. 1:**
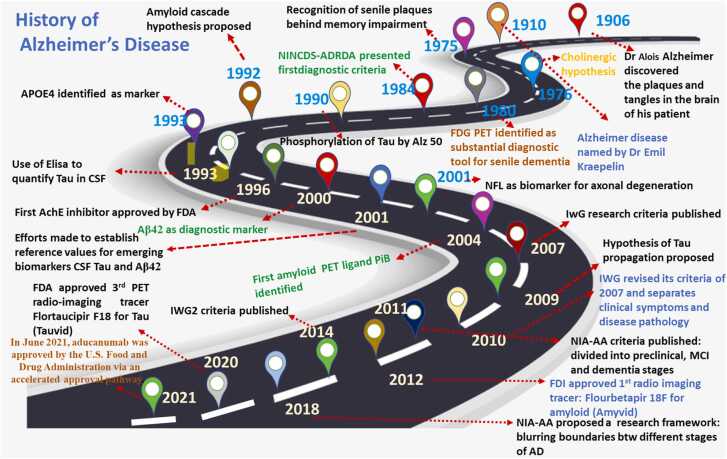
The history of AD and several advances in pathogenesis research, imaging technology advancements, and diagnostic paradigm shifts

**Fig. 2:**
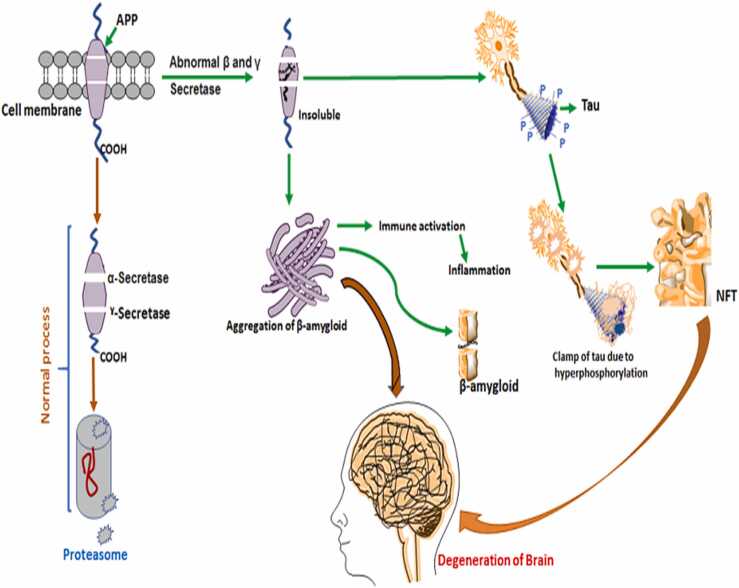
Pathophysiology of Alzheimer's disease.

Since its discovery in 1906, researchers have worked to better understand the neuropathology of Alzheimer's Disease (AD) and create diagnostic techniques for successful therapeutic strategies to slow or stop the disease's progression. The increased prevalence of AD has made it amongst the most slowly progressive elderly diseases that affect the life quality of patients, and it is the fifth leading cause of death globally (Kunkle et al., 2021)). The earliest and most common symptoms of AD are; squandering the short-term memory, with steadily emerging cognitive-behavioral dysfunction, ultimately leading to progressive neurodegeneration (Kurth et al., 2019). Deposition of amyloid-β plaques (Aβ), hyperphosphorylation of tau, and initiation of several neurometabolic responses in the brain, followed by the loss of neuronal and synaptic functions, and also the impairment of learning and memory functions, are the central neuropathological characteristics of AD. The accumulation of plaque has been initially started in the medial temporal lobe, mainly the hippocampus and entorhinal cortex, before the appearance of behavioral symptoms. These changes have been known to play a part in decreasing the pattern of learning and memory processes and impairing cognitive properties (Hartman and Ross, 2018). In cerebrospinal fluid (CSF), a disproportion of Aβ to Aβ42 protein is a key criterion to confirm the pathology of AD clinically (Kim et al., 2019). Even though the symptoms of diverse neurodegenerative dementias are often similar, each is usually distinguished by the deposition of its characteristic protein isoform in specific brain areas. Positron Emission Tomography (PET), Cerebrospinal Fluid (CSF), and blood are the most commonly used methods in which biomarkers are studied. CSF can immediately reveal the biochemical changes in the brain, nevertheless, the invasiveness of lumbar puncture restricts its application in ordinary medical care. Imaging biomarkers are the most commonly used diagnostic tool in clinics, but they, too, offer a risk of radiation exposure and impose a financial burden on patients (Hampel et al., 2018). The Alzheimer's Precision Medicine Initiative formed an international interdisciplinary workgroup in 2016 to prioritize research for identifying possible blood-based biomarkers, keeping in mind the difficulties in detecting Alzheimer's disease in general practice (Ahmed et al., 2021). Data from numerous studies is being collected to set cut-off values for biomarkers and standardize measuring methodologies to enhance the diagnosis of Alzheimer's disease to produce consistent results throughout time (Fig. 1). Aβ is formed due to the progressive breakdown of amyloid precursor protein (APP) by the activation of the β-secretase enzyme, 'β-site amyloid precursor protein cleaving enzyme 1′ (BACE 1), and the γ-secretase in the amyloidogenic pathway (Fig. 2) (Kim et al., 2019). Amyloid-beta (Aβ) peptides are the extracellular plaques, which are mainly cleaved from amyloid precursor proteins (APP) by a complex of enzymes called β-secretase and γ-secretase enzymes. The increasing concentration of these extracellular Aβ monomers gradually increases by their polymerization followed by aggregation and ultimately result in dense-core amyloid plaques (D'Onofrio et al., 2017). APP is a transmembrane protein that plays a substantial part in the development and growth of neurons and anterograde axoplasmic trafficking (Kienlen-Campard et al., 2008). The Aβ peptides which are produced after the cleavage at the γ site undergo the process of oligomerization and consequent fibrillization for the formation of distinctive plaques of β-amyloid in AD (Kutovyi et al., 2020). On the other hand, the α-secretases start the non-amyloidogenic pathway which cuts the APP at the extracellular domain of α-site and produces the soluble fragment of APP. This proteolytic processing of APP by the α-secretase enzyme results in the production of soluble fragments of sAPPα, in contrast to sAPPβ. Both these pathways are active in the physiological system, but the mechanism of the non-amyloidogenic pathway of processing of APP is highly favored thus, signifying that the cleavage of APP by α-secretase is decisive to the regular function of the brain. More than 90% of APP are cleaved by α -secretase in the normal physiological state, and the remaining 10% of APP is being cleaved and processed by β and gamma-secretases, thus producing Aβ42 and Aβ40 insoluble peptides (Bettens et al., 2010). The clearance and/or aggregation of Aβ is too dependent on the allele of the *APOE* gene which codes for apolipoprotein E, which functions as trafficking of lipid. Among the four alleles of the *APOE* gene, the *APOEε4* allele is existing in 14.5% and 40% of the overall population and with late-onset AD, while rare *Apoε2* allele (6.4% of the population) may have a protective role for AD (Ostendorf et al., 2020). The detailed process of *APOE* in the onset of AD is still not well stated and it needs more widespread mechanistic and biochemical research to unveil its role. Moreover, the recent studies have also confirmed that neuroinflammation, synaptic dysfunction, mitochondrial dysfunction, microglial activation, astrogliosis, loss of neurons, damage in the permeability of blood brain barrier, viral, bacterial and intestinal infections have been substantial factors which are responsible to pathophysiology of AD (Hartman and Ross, 2018).

(modified from Ahmed, et al., 2021).

The existing therapeutic drugs for the treatment of Alzheimer's disease such as Rivastigmine and galantamine increases the level of cholinergic system in brain of AD patient but these drugs also have adverse side effects (Wightman, 2017). Several bioactive phytochemicals, including vitamins (e.g., folic acid and tocopherols) and many additional organic compounds (e.g., alkaloids, terpenes, phenols, flavonoids and organosulfur) can dysregulate the main characteristics of the AD disease pathways. The substantial mechanisms for their ability to effects those pathways are their antioxidant properties, enhance the immune system in brain by releasing anti-inflammatory cytokines and also modulates the toxicity and regulation of Aβ in brain of AD patient (Kim et al., 2010).

## 2 Tau and AD

Tau is an unstructured protein and in an adult human's brain, it shows its expression in six different isoforms which occurs due to the alternative splicing of the Microtubule Associated Protein Tau (*MAPT)* gene. Tau protein displays a variety of functions in normal conditions, of which the tendency to regulate the microtubules inside the neuronal axons is the most well studied (Boutajangout et al., 2004). Tau isoforms consist of 3 or 4 microtubule-binding repeats (MTBRs; 3 R or 4 R) that facilitate tau-to-microtubule binding (Goode et al., 2000). Besides, the tau comprises 0–2 acid domains (0–2 N) in the N-terminal of molecules and an intrinsic proline-rich domain in between the N-terminal sequences and the MTBRs (Wang et al., 2004). Abnormal tau protein misfolding results in the development of beta-sheet fibrils that aggregate within the cells of the central nervous system (CNS), which cause neurodegenerative diseases and is called Tauopathies (Kovacs, 2018). Tau proteins play a vital role in the physiological functioning of neurons because they cause the polymerization and stabilization of microtubules, transportation of various enzymes and other organelles along the cellular cytoskeleton, and also promote the growth of neurons (Kovacs, 2018). Tau undergoes various types of post-translational modifications due to the presence of more than 30 different substantial phosphorylation sites. This happens because of the large amount of significant serine and threonine phosphate accepting residues. The various post-translational modifications include phosphorylation by several kinases such as GSK-3β, AMPK, and JKN, (Martin et al., 2011). A large number of abnormal fragments are formed due to the hyperphosphorylation of tau proteins, which aggregates and form tau oligomers and eventually leads to the paired helical or straight filaments which start to constitute the (NFT) Neurofibrillary tangles (Fig. 2). These NFT reduce the affinity of tau proteins towards microtubules and lead to neuronal toxicity. It is still unknown that the toxicity occurs either due to phosphorylation of tau at specific amino acid sites or the phosphorylation of a certain amount of tau must be phosphorylated before the onset of several pathological effects (Iqbal et al., 2010). The neurotoxicity occurs through two different major pathways: toxic loss of function, in which the destabilization of microtubules occurs due to the loss of physiological function of tau protein, and toxic gain of function, in which extremely phosphorylated tau exhibit its toxic effects in neurons (Ballatore et al., 2007). Besides, some in vitro studies have shown that prefibrillar and soluble tau oligomers have more toxic features relative to higher-order tau aggregation.

## 3 Hypothesis for the amyloid cascade

The Aβ peptide is produced by the breakdown of amyloid precursor protein (APP) and it is a type I transmembrane glycoprotein of 695–770 amino acids (Sajad et al., 2022). α-secretase which is an extracellular protease cleaves the APP near the membrane. This cleavage releases an extracellular soluble fragment called sAPPα (Fig. 2). An aspartyl protease called β-secretase-1 (BACE1) also cleaved the APP, forming sAPPβ, which is an extracellular soluble fragment, and a cell-membrane-bound fragment (C99). An enzymatic complex of four proteins (anterior pharynx defective presenilin, nicastrin, and presenilin enhancer 2), collectively called γ-secretase, breaks the C99 fragment in the membrane. Presenilin acts as the catalytic subunit of the γ-secretase complex and thus it could be encoded either by the *PSEN1* or *PSEN2* gene. This γ-secretase complex acts on Aβ and an intracellular peptide are released by this cleavage thus called the amyloid intracellular domain. There are different lengths of Aβ, the most abundant form consisting of 40 amino acids (Aβ1–40) and a less soluble form of 42 amino acids (Aβ1–42). the aggregation of Aβ form oligomers, protofibrils, fibrils, and eventually the formation of plaques occur, which is one of the defining characteristics of AD pathology. It is believed that the accumulation of Aβ in the brain is the initial event of the AD phase. The accumulation of Aβ begins in the hippocampus and entorhinal cortex region. Furthermore, the hyperphosphorylated intracellular deposition of tau protein leads to the formation of neurofibrillary tangles (NFTs), which contributes to incremental changes in the cytoskeleton and also disrupts the axonal transport. The hypothesis, that the accumulation of Aβ in the pathogenesis of AD as a core event was originally suggested by three separate groups in 1991. The 'amyloid cascade theory' was formally suggested one year later by Hardy and Higgins (Ricciarelli and Fedele, 2017). Initially, this hypothesis specified that the hyperphosphorylation of tau protein, the formation of neurofibrillary tangles, loss of synapse, death of neurons, and cognitive impairment are driven by the deposition of Aβ in the brain. This hypothesis was later also confirmed by several studies that the autosomal mutation in APP genes leads to the progression of AD and this further leads to the Aβ production in large amounts thus facilitating the aggregation of Aβ and its deposition. Transgenic mice expressing variants of APP or presenilin protein carrying human familial AD-associated mutations have increasingly developed Aβ plaques and memory defects in the brain, additionally strengthening the principle that the accumulation of Aβ can cause AD (Karran et al., 2011). Accumulation of Aβ in the brain occurs due to abnormal clearance (Jonsson et al., 2012) and enhanced activity of BACE (Panza et al., 2019) in the late-onset AD. Accumulation of Aβ is also being associated with the apolipoprotein E * ε4 (APOE*ε4) allele, which is the genetic risk factor for the onset of late sporadic AD. APOE which is a fat-binding protein is primarily produced by the astrocytes in the CNS and its primary function is to transports cholesterol through *APOE* receptors to neurons. The three main alleles of *APOE* i.e., APOE* ε2, APOE* ε3, and APOE* ε4, have made the polymorphic nature of this gene. AD risk is up to 15 times greater in the individuals with two APOE* ε4 alleles as compared to APOE* ε3 carriers and the allele APOE* ε2 may give defense against AD. Concerning AD risk, the APOE* ε3 allele seems to be neutral. The various *APOE* proteins display different abilities to initiate Aβ clearance, with the most effective being *APOE2* and the least effective being *APOE4* (Liu et al., 2013, Williams et al., 2020). In plaque-free aged people without dementia, the APOE* ε4 allele is strongly associated with longitudinal abnormal aggregation of Aβ. Also, the carriers of APOE* ε2 are protected against this longitudinal accumulation of Aβ (Lim and Mormino, 2017).

In the people of older age groups, the consequences of AD are more insidious and progressive. The formation and deposition of amyloid plaques (extracellular) and neurofibrillary tangles (intracellular) are two main hallmarks in the brain of the AD patient. The activated microglia surrounding the senile plaques along with higher concentrations of different components of the complement system, chemokines, cytokines, and free radicals have always been observed (Sansores-España et al., 2021). These studies directed to the knowledge that neuroinflammation can cause a self-propagating toxic process where several factors including the misfolding of proteins and their aggregation, abnormal cellular components, damaged neurons, and abnormal synapses induce microglia to release inflammatory cytokines, which in turn intensify the β-amyloid deposition and neuronal injury (Leyns and Holtzman, 2017).

## 4 Inflammatory mechanism and mitochondrial dysfunction

Several studies have pointed out the association of neuroinflammation in the development of different neuropathological variations in AD. The progression of AD is directly connected to inflammatory processes (Friedman et al., 2015). Several studies have shown that even during the emergence of acute and chronic inflammatory progressions Tau pathologies are dramatically increased (Guedes et al., 2018, Stancu et al., 2014). The role of inflammation tends to have a double purpose in the brain, i.e., neuroprotective function in an acute phase of the reaction, but in the chronic stage, it becomes harmful. The progression of those inflammatory processes is regulated by microglial clusters around the Amyloid-beta plaques, thus releasing several pro-inflammatory cytokines due to the activation of microglial cells leads to the production of neurofibrillary tangles (Wei et al., 2019). It is reported that the accumulation of β-Amyloid plaques and abnormal Tau protein inhibits the function of mitochondria especially by diminishing the mitochondrial oxidative metabolism (Cabezas-Opazo et al., 2015). Further studies associated with the production of Amyloid beta peptides also relate that these peptides could be directly toxic to the neurons of the mitochondrial region (Ganguly et al., 2017). Several proinflammatory mediators and other toxic products, together with cytokines, nitric oxide, and reactive oxygen species are chronically activated and released by microglial cells. The increased level of several cytokines like interleukin 1β (IL-1β) has been studied due to the deposition of Aβ in the cerebral region in deceased patients suffering from head trauma. This increased production of such molecules is responsible for the production of APP and Aβ (Frost and Li, 2017, Klomparens and Ding, 2020). Moreover, this raised production of IL-1β has also been discovered to enhance the generation of some additional cytokines, such as IL-6, which further activate *CDK5*, a kinase that has been identified to be linked with hyper-phosphorylation of tau (Yang, 2019). In AD conditions, neuroinflammation serves a primary role in worsening the load of Aβ and hyperphosphorylated tau thus, signifying that these two conditions could be responsible and leading factors that have an association between different pathologies of AD. The raised immune response after all these conditions by the macrophage immune cells (microglia) present in the brain is now a core aspect in AD study.

### 4.1 NF-κB signaling controls neuroinflammation in AD and facilitates synaptic dysfunction

There has been a close association between neurons degeneration, synaptic dysfunctions due to the activation and increased expression of nuclear factor-κB (NF-**κ**B). NF-**κ**B is a heterodimer (p50 and p65 subunits) as it regulates the activation and generation of various inflammatory cytokines. It causes the phosphorylation and releasing of its cytoplasmic inhibitory protein inhibitor of ϏB (IϏB) and nuclear translocation of transcription factors. Large numbers of proinflammatory cytokines are releases due to the activation of NF-**κ**B in glial cells during the pathophysiology of AD (Seo et al., 2018). The NF-κB like other transcription factors substantially regulates the inflammatory and several other pathological processes (Srinivasan and Lahiri, 2015). Since then, NF-κB together with its family members, p50, p52, p65 (Rel-A), Rel-B, and c-Rel proteins, have been discovered to present almost in all forms of animal cells and are involved as a key agent against various stimuli (Srinivasan Lahiri, et al., 2018). Neurotoxicity, oxidative stress, and inflammation are caused by Aβ, which increases progressive neurodegeneration, resulting in nerve damage and neuronal death (Caruso et al., 2019). NF-κB (p65) upregulates the transcription of the human *BACE1* gene and promotes the processing of β-secretase by β-amyloid precursor protein to produce Aβ (Fiebig et al., 2014, Wang et al., 2017). In the neuronal and glial cells, NF-κB also increased its activity near the site of Aβ plaques (Shi et al., 2017). This activity of NF-κB remains enhanced and associated during the inflammatory condition and shows its predominant expression in peripheral blood mononuclear cells (PBMCs) and in the brain of AD patients, which is proposed as a significant marker for the progression of AD (Juźwik et al., 2019). P^65^ translocate to the nucleus by the activation of inflammatory signals. It can be said that the activation and recognition of NF-κB in the Central Nervous System initiates the inflammatory reactions, which consecutively enhance the expression of various pro-inflammatory genes (Lukiw, 2016). Subsequently, NF-κB appears to cause neuroinflammation, which leads to the production of oxidative stress and various other cascading conditions that allows Aβ42 to activate the expression of the inducible nitric oxide synthase gene (iNOS) and the development of nitric oxide, which is wholly reliant on NF-κB (Lukiw, 2016). These findings demonstrate that in microglia, NF-κB is not "protective" but rather neurotoxic (Xiao et al., 2018). NF-κB is present in the cytoplasmic regions of dendritic and somatic neurons and its activation pathway is localized in the synapses. In the hippocampal region, NF-κB is present in an abundant concentration in postsynaptic regions of the dendritic spines of the neurons (Dresselhaus and Meffert, 2019). The postsynaptic density protein-95 (PSD-95) is a vital marker gene of NF-κB, and the p65 subunit has been found to regulates both the development and morphology of dendritic spines and excitatory synapses in the neurons of hippocampal regions, which are necessary for learning and memory (Dresselhaus et al., 2018). However, available studies concluded that it is not possible to disregard the neuroprotective ability in response to activation of NF-κB. After oxidative stress exhibited by TNF-alpha-mediated ITSC-derived neurons, the expression of sex-specific NF-κB-p65 target genes relies on TNF-alpha-mediated neuroprotection (Ruiz-Perera et al., 2018). The neurons of the hippocampal region displayed increased excitotoxicity in mice due to the lack of the p50 subunit of NF-κB (Bellucci et al., 2020). Endogenous stimulation of NF-κB by Aβ reduced the activity of *BACE1*, γ-secretase, and *βAPP* under physiological conditions, which sequentially inhibited the development of Aβ (Wang et al., 2017). Moreover, only low Aβ concentrations have been reported to cause an NF-κB-dependent protective phenotype (Kaltschmidt et al., 1999). The signaling pathway of NF-κB can therefore unique, early and the main neuroprotective pathways to detect in patients with AD (Table 1).

**Table 1:** Depicts the signaling pathways of NF-κB involved in the mechanism of neuroinflammation and synaptic failure.

Induced model	Object	Target	Main effect	Reference
Aβ induced	Astrocytes	↑NF-κB, iNOS, NO	Aβ stimulates the expression of iNOS	(Akama et al., 1998)
NF-κB	N2a and SHSY5Y cells, APPswe/ PS1dE9	BACE1, APP, Aβ	NF-κB dependent regulation of BACE1 transcription by BAG-1 M	(Shi et al., 2017)
TNF-α, IL-1, IL-6	N2a neuroblastoma cells, mice cortical neurons	↑APP	inhibition of β-secretase by heparan sulfate is reduced	(Fiebig et al., 2014)
ROS→NF-κB	BV-2	↑iNOS, COX-2	Neurotoxic effect on BV-2 primarily through MAPKs and NF-κB pathways facilitated by ROS	(Xiao et al., 2018)
NF-κB (p65)	Human, cells	↑BACE, APP	Promote APP β-secretase processing to produce Aβ	(Chen et al., 2012)
NF-κB p50 subunit	p50 KO	Induced LTP	Deficiency of the NF-κB p50 subunit led to substantial reductions in late LTP	(Oikawa et al., 2012)
↑NF-κB and C3	Astroglial, APP/PS1, GcKO	↑Aβ	Impaired Aβ clearance due to enhanced astrocytic NF-κB and C3 signaling, thus promotes glial inflammation in the brain	(Lian et al., 2016)
NF-κB	Human, 3xTg-AD	↑BACE, APP	Dysfunction of synaptic plasticity due to the activation of NF-κB	(Sarkar et al., 2016)

### 4.2 The function of oxidative stress and other factors in the development and pathogenesis of AD

The role of ROS-induced oxidative stress in the progression and development of AD has been extensively studied so far. (Cenini et al., 2019), and it has been recognized and debated as a substantial factor in the pathogenesis of AD for over twenty years, with a variety of review papers accentuating the crucial function of ROS in the pathology of AD (Zhang et al., 2015). Substantial oxidative damage to neuronal tissue is frequently present in the atrophied brains of AD patients (Huang et al., 2016). Highly unstable free radicals, for instance, reactive oxygen species (ROS) are the central reactive molecules that participate as an oxidant or reductant in the redox reactions which is attributed because of an unpaired electron present in its atomic orbital (Kregel and Zhang, 2007). The oxidative stress leads to the progression of AD in three major ways: (i) the macromolecule peroxidation, (ii) Aβ metal ion redox potential, and (iii) the mitochondrial dysfunction, all of which affect cell homeostasis, the generation of ROS, and the up-regulate the generation of Aβ and p-tau. Osmotic stress has been studied to increase the production of iron-redox-active state in Aβ and NFTs. Large number of reactive oxygen species are formed due to the abnormal function of Amyloid-β-precursor proteins that further leads to the dysfunctions of mitochondrial genome and substantial metabolic enzymes. These ROS also cause the severe pathological conditions in brain dur to the damages of macromolecules such as damages in DNA, RNA, Lipids and Proteins. Thus, there is an imbalance between the defensive system of cells and the generation of ROS, which in turn lead to cognitive dysfunctions and neurodegeneration (Reza et al., 2018). Moreover, this imbalance also stimulates the expression of several downstream signaling molecules, like mitogen associated kinases (MAPKs) and Protein Kinase-C (PKC), which in turn causes the enhanced expression of proinflammatory cytokines and nuclear translocation of NF-ϏB (Srinivasan et al., 2018).

#### 4.2.1 Lipid peroxidation

Disruption of DNA protein cross-links, lipid peroxidation, and oxidative damage of the cells have been studied to be involved in the progression of aging and multiple chronic disorders, together with arthritis, diabetes, tumors, cardiac disorder for instance Atherosclerosis and heart disease, and other degenerative disorders, like muscular dystrophy, AD and Parkinson's disease (Lobo et al., 2010). Double bond peroxidation occurs in the polyunsaturated neuronal lipids that lead to the development of by-products of biochemically active lipids, particularly malondialdehyde, F2-isoprostanes, and 4-hydroxy-2, 3-nominal (HNE) (Bradley-Whitman and Lovell, 2015).

These molecules are highly reactive and are capable of inducing tau phosphorylation and instability, interruption in the pathway of intracellular signaling of Ca^2+^, and activation of the apoptotic cascade (Butterfield and Halliwell, 2019). Moreover, oxidative modification of lipids due to the ROS-induced oxidative stress and increased production of HNE were studied in pyramidal hippocampal cells and neurons that contain NFTs (Barrera et al., 2018). The mitochondrial and nuclear nucleotides have a higher susceptibility to ROS-induced oxidative damage via carboxylation, nitration, and hydroxylation, (Lovell and Markesbery, 2007). Such epigenetic regulations are responsible for the onset of variations in the binding behavior of the transcription factor, disruption in the crosslinking pattern of DNA and proteins, and the initiation of mutagenic characters (Dizdaroglu and Jaruga, 2012). Nucleotide oxidation creates biomarkers that can be studied to assess the degree of damage caused by ROS, particularly 8-hydroxydeoxyguanine (8-OHdG), which have been identified as a key player in the formation of Aβ and hyperphosphorylation of tau protein in the frontal, parietal, and temporal lobes (Gabbita et al., 1998, Dizdaroglu and Jaruga, 2012). Eventually, ROS-induced oxidative damage has been shown to speed up the oxidation of Glycated proteins that are produced by protein and saccharide reactions (Münch et al., 1998). These advanced glycation end products (AGEs) are effective proinflammatory and neurotoxins agents, since they have the capability of binding to the receptor of the advanced glycation end product (RAGE), and further cause the increased production of several inflammatory molecules such as interleukin-1, nitrous oxide, COX2, and TNF-α, that ultimately cause damage to neuronal tissue in chronic conditions (Moldogazieva et al., 2019). Besides, the physiological tau proteins can transform into AGEs after being glycated thus, hindering its binding capability to microtubules and the development of higher-order fibrils. In the same way, the process of glycation and formation of AGE from the monomers of Aβ have been proved to cause oligomerization as well as the formation of complex plaque due to the enhanced Aβ cross-linking (Guan et al., 2019).

#### 4.2.2 Mitochondrial dysfunction

Mitochondria are intracellular, maternally inherited organelles, play a substantial role in the metabolism, and are the second signaling messenger in the process of programmed cell death. The synthesis of oxygen‐dependent ATP is regulated by enzyme complexes that constitute the mitochondrial electron transport chain (ETC) in oxidative phosphorylation. Mitochondriopathies is characteristically considered as a mitochondrial dysfunction. These can be inherited genetically mitochondrial proteins encoded by mitochondrial DNA [mtDNA] or nuclear DNA) or acquired. Typically, Mitochondriopathies shows heterogeneous nature, due to the involvement of multiple organs Rahman et al. (2020a).

## 5 Aβ and p-tau upregulation and toxicity

Both tau phosphorylation and Aβ generation are upregulated by oxidative stress. The proteolytic activity of BACE1 and the expression of mRNA are enhanced and associated with increased production of several oxidative biomarkers, e.g., HNE during hypoxia and other oxidizing agents (Guglielmotto et al., 2009). The signaling of BACE1 is upregulated due to the binding of AGEs to RAGE, together with activation of signaling pathway NF-κB, which causes the transcription and synthesis of *BACE1* along with the generation of Aβ (Kuhla et al., 2015). The enhanced processing of Aβ and aggregation of the peptide may be affected by the lipid deposition surrounding the receptors of nicotinic acetylcholine which result in their function modification (Fabiani and Antollini, 2019). Likewise, the binding of AGEs to RAGE decreases the concentrations of lithium chloride that activates the GSK-3β enzyme which ultimately causes phosphorylation of tau (Cai et al., 2016). Increased levels of GSK-3β enzyme and p-tau have also been observed in cortical neurons of experimental rats after the treatment with hydrogen peroxide (H2O2) in PC12 cell culture (Wang et al., 2020). Finally, it was reported that HNE activates both p38 MAP and JNK1 kinases that cause accelerated tau phosphorylation during enhanced oxidative stress levels (Li et al., 2018a). The generation and oligomerization of Aβ and p-tau have been increased due to the production of ROS and oxidative stress, which result in abnormal function due to migration of molecules towards dendritic spines, deterioration of the function of receptors located at the postsynaptic membrane. As a result of which the collapse of the dendritic spine and synapses loss occur due to the activations of several proinflammatory cytokines (Nisbet et al., 2015). The oligomers of Aβ act on extra-synaptic N-methyl-D-aspartate receptors (NMDAR), beginning the invasion of Ca2 + into the neuron thus cause the activation of GSK-3β and AMPK (tau protein kinases). Hyperphosphorylation of tau also leads to the binding of p-tau to Fyn kinase, resulting in the movement of p-tau to the neurons of dendritic regions (Nisbet et al., 2015).

**Fig. 3:**
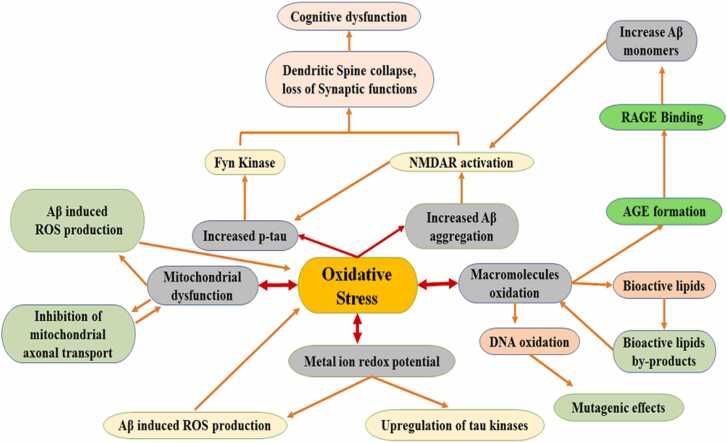
The interconnection between oxidative stress (OS) and Alzheimer's Disease (AD).

Also, Fyn and Aβ-bounded p-tau shows synergistic properties, leading to reduced levels of NMDARs on the surface and subsequently result in the death of the dendritic spine and synaptic failure. These conditions further cause the inhibition of Long-term hippocampal properties, thereby impacting the memory (Musardo and Marcello, 2017). Finally, striatal-enriched protein tyrosine phosphatase (STEP) protein is activated by Aβ which in turn inactivates the Fyn that causes the collapse of the dendritic spine due to a series of downstream reactions (Fig. 3) (Amar et al., 2017). A positive feedback loop is produced by oxidative stress, which increases the production of ROS, generation of Aβ, and phosphorylation of tau that leads to additional oxidative stress. Eventually, this neurodegeneration pathophysiological pathway leads to severe neuronal damages (Sajjad et al., 2019). It is also clear that the interaction between increased oxidative stress, formation of Aβ, and hyperphosphorylation of tau is important for studying clinical effects in AD patients.

## 6 Role of phytochemicals in the prevention of Alzheimer's disease

Phytochemicals are the secondary metabolite and have significant biological and health-promoting effects. By reducing or repairing cellular dysfunctions, phytochemicals have been found to inhibit the progression of several chronic diseases, such as cancer, urolithiasis and cardiovascular disease (Sajad and Thakur, 2020). Although polyphenolic compounds are the most common form of secondary metabolites and several in vitro and in vivo studies have shown that these phytoconstituents have substantial antioxidant activity that prevents the progression of diseases. Catechins, resveratrol, berberine and curcumin are among the phytochemicals that are gaining popularity, according to epidemiological studies that show a link between their consumption and the prevention of Alzheimer's disease (Singh et al., 2008). These phytochemicals target the intracellular and molecular markers of diseases and inhibit or upregulate their expression that reduces the initiation or severity of AD.

### 6.1 Curcumin

**Fig. 4:**
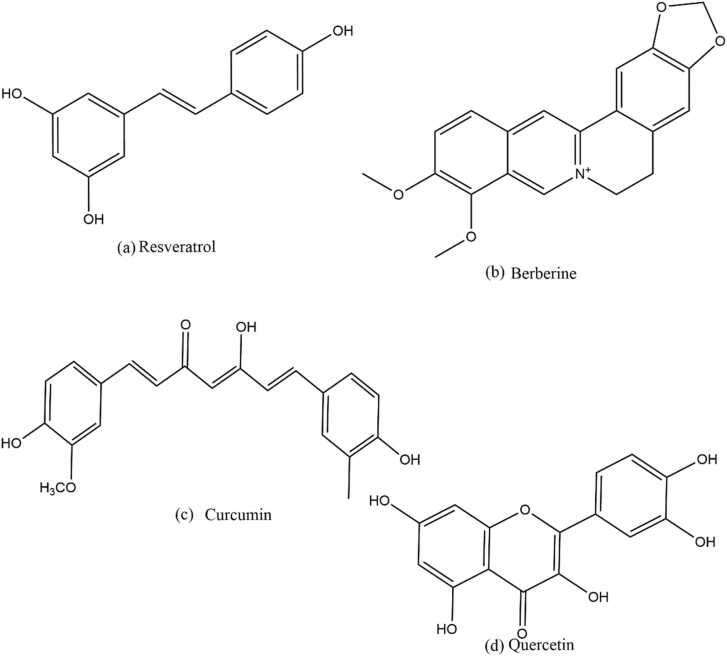
Structures of phytochemicals.

Curcumin (C_21_H_20_O_6,_ Fig. 4), found particularly in turmeric rhizome (*Curcuma longa*), is used as a spice, flavor enhancer as well as a food preservative and. It was recently discovered that the pervasiveness of Alzheimer's disease in Indian adults aged 70–80 is 4.4 times lesser than in the United States, indicating that a curcumin-rich diet may be substantially accounted for the lower risk of AD (Das and Devi, 2019). Curcumin possesses anti-oxidative, anti-cancer, anti-inflammatory and anti-amyloidogenic properties, thus it can prevent the prevalence of AD (Ringman et al., 2005). Curcumin has been studied to inhibit the production and accumulation of Aβ plaques, which is a defining feature of AD. The treatment of intragastric curcumin to AD model, lowered the severity of the disease by inhibiting the expression of *BACE1*. It also reduce the degeneration of synapse and enhance the spatial learning and memory pattern (Zheng et al., 2017). *Presenilin-1* (*PS-1*)*,* a protein in the -secretase complex and a substrate for glycogen synthase kinase-3β (GSK-3β), is another enzymatic target for the synthesis of Aβ. Both -secretase and GSK- 3β have been linked to the production of Aβ. Curcumin treatment of human neuroblastoma SHSY5Y cells resulted in a significant reduction in Aβ production. The expression of both PS-1 and GSK-3β protein reduces in a dose- and time-dependent manner after treatment thus, implying that curcumin reduced Aβ synthesis via inhibiting GSK-3β dependent PS-1 activation (Zhang et al., 2011). Curcumin also plays a role in the removal and inhibit the formation of tau tangles and neurotoxicity. *BCL2* associated athanogene 2 (*BAG2*) is a molecular chaperone that transports tau to the proteasome, where it is degraded (Carrettiero et al., 2009). *BAG2* was significantly increased and hyperphosphorylated tau levels decreased in rat primary cortical neurons after treatment with curcumin (Patil et al., 2013). It has also been studied that curcumin shows neuroprotective properties by reducing or preventing the generation of ROS, nitric oxide free radicals and by reducing the neuroinflammation. It diminishes the oxidative stress in the neuronal region by upregulating the expression of certain proteins which protect the cells along with the expression of several antioxidant enzymes. Moreover, curcumin also induces the substantial increase in the expression of intracellular glutathione by generating the transcription factors like electrophilic response element (EpRE) and 12- tetradecanoate 13-acetate (TPA) (D'Onofrio, et al., 2017).

### 6.2 Resveratrol

Resveratrol (C_14_H_12_O_3_) is a polyphenolic phytoalexin (Fig. 4) present in red wine, grapes, and berries. It has varieties of pharmacological, cellular and biological effects (Tian and Liu, 2020). Several epidemiological studies have been found an inverse association between wine consumption and the risk of Alzheimer's disease. Thus, leading to conjecture that resveratrol may play a therapeutic effects in Alzheimer's patients (Rahman et al., 2020b). Resveratrol promotes non-amyloidogenic cleavage of the amyloid precursor protein. It also improves amyloid beta-peptide clearance and decreases neuronal damage. During the last few years, the majority of experimental research on AD and resveratrol has been conducted in a variety of species, both in vitro and in vivo. The use of polyphenolic constituents from grape seed extract (GPSE) reduced Aβ peptide oligomerization and reduced cognitive decline in Tg2576 mice (Wang et al., 2008) The quantity of activated microglia drops significantly in resveratrol-treated *APP/PS1* mice, indicating that resveratrol, regardless of its effects on amyloid formation, reduces inflammation (Jeon et al., 2012). Glutamate induced the synthesis of monocyte chemical protein1(MCP-1) in 10-day Sprague–Dawley rat pups. Resveratrol inhibited glutamate-induced ERK activation, resulting in lower interleukin-1 (IL-1) expression and downregulation of MCP-1 in the hippocampus (Lee et al., 2010). It protects the high fat diet (HFD)-induced memory loss. Resveratrol also reduces the amyloid load exacerbated by high fat diet (HFD) in 5XFAD and protected both wild type and 5XFAD strains against HFD-induced tau pathology. Resveratrol inhibited the HFD-induced amyloidogenic processing of the amyloid precursor protein in both strains, as well as restoring abnormally high levels of the ubiquitin-proteasome system's proteolytic activity, implying the presence of a compensatory mechanism to counteract the accumulation of aberrant proteins (Sarroca et al., 2021). By stabilizing protein-substrate linkages, resveratrol promotes SIRT1 functioning (Lin et al., 2014). In addition to increasing SIRT1 mRNA expression, resveratrol also increases SIRT1 protein expression and activity(Cheng et al., 2012). SIRT1 is thought to be a crucial protein involved in numerous resveratrol actions, and its activation is thought to be the basis of resveratrol-mediated protection. In human-derived neuroblastoma cell lines, resveratrol inhibits apoptosis, inhibits the inflammatory response, reduces oxidative stress, and promotes autophagic flux stabilization via the SIRT1 signaling pathway ( Li, et al., 2019 and Wang et al., 2014).

### 6.3 Berberine

Berberine (C_20_H_18_NO_4_ +, Fig. 4) is a bitter-tasting yellow plant alkaloid that has been used in Chinese and Ayurvedic medicine for more than 3000 years. Berberine's sedation-inducing pharmacological impact on the neurological system was originally described in the 1970 s (Shanbhag et al., 1970). Berberine's therapeutic potential has been studied in a variety of neurological disorders, including cerebral ischemia, Alzheimer's disease, Parkinson's disease, depression, anxiety, Huntington's disease, epilepsy, and convulsions. Berberine has been found in reducing Alzheimer's disease pathogenesis by inhibiting hyperphosphorylation of Tau protein and Aβ formation. Berberine inhibits the synthesis of Aβ40/42 via stimulating the extracellular signal-regulated kinase 1/2 signaling pathway, which inhibits beta-secretase expression (Durairajan et al., 2012). Furthermore, researchers have discovered that berberine inhibits the four major enzymes involved in the pathogenesis of Alzheimer's disease: acetylcholinesterase, butyrylcholinesterase, monoamine oxidase A, and monoamine oxidase B (Ji and Shen, 2012). Furthermore, berberine can also attenuate Tau hyperphosphorylation, that could enhance the activation of the phosphatidylinositol 3-kinase/protein kinase/glycogen synthase kinase 3 pathway, which increases the activity of protein phosphatase 2 A and reverses GSK-3 activation (Yu et al., 2011). According to the cholinergic hypothesis, reduced cholinergic activity is linked to AD symptoms, and improving cholinergic activity will reduce AD symptoms. The key enzyme for acetylcholine breakdown is cholinesterase (ChE), and inhibiting it causes an increase in acetylcholine levels in the brain. As a result, cholinesterase (ChE) inhibitors have been the focus of numerous anti-AD pharmaceutical research to alleviate cognitive symptoms (Cummings, 2000). Several experiments have been conducted to see how berberine affects ChE activity. In ethanol-treated rats, berberine (25–100 mg/kg) reduced oxidative stress and ChE activity (Yu, et al., 2011). A one-month treatment with berberine had a similar promising impact on streptozotocin-induced memory impairment in rats (de Oliveira et al., 2016). Berberine (100 mg/kg) administration during training trials increased learning and memory while lowering oxidative stress, hyperglycemia, and ChE activity (Bhutada et al., 2011). In an AD transgenic mice model, berberine has been found to reduce β-amyloid pathology and cognitive impairment (Huang et al., 2017). The levels of extracellular and intracellular A1–42 was reduced after berberine treatment, which was mediated by increased the autophagy activity.

### 6.4 Quercetin

Quercetin (C_15_H_10_O_7_, Fig. 4) is a flavonoid with significant therapeutic and pharmacological properties. The neuroprotective properties of quercetin have been investigated extensively. It reduces cell toxicity caused by oxidative stress in neurons at low micromolar doses. It inhibits neuroinflammation by inhibiting pro-inflammatory cytokines including NF-kB and iNOS while also promoting neuronal regeneration. By stimulating the PKD1-Akt cell survival signaling axis, quercetin protects the mitochondrial failure and progressive dopaminergic neurodegeneration (Ay et al., 2017). In HT22 cells, it uses MAPKs and the PI3K/Akt/GSK3 signaling pathways to reduce the hyperphosphorylation of tau proteins (Jiang et al., 2016). *In vivo* studies have demonstrated that quercetin reduces the generation of nitric oxide, iNOS gene expression in microglia, and the production of inflammatory cytokines such as interleukin-1 (IL-1), tumor necrosis factor (TNF-), COX-2, IL-6, and IL-12 in activated macrophages, along with the reduction in expression of cytokine. Quercetin also suppresses TNF-production and decreases JNK/Jun phosphorylation in rats, protecting neurons from LPS-induced inflammation (Qureshi et al., 2011). Moreover, several neuroprotective approaches such as reducing neuroinflammatory mechanisms, neuroreparative strategies, antioxidant methods, hormonal strategy, and oxidative stress. Among all the neuroprotective methods, the source of phytochemicals are the emerging and substantially valuable sources for preventing and treating the pathophysiology of AD. Some phytochemicals which have significant drug like behaviors and have been studied for the neuroprotective effect are listed in Table 2.

**Table 2:** phytochemicals depicting their diverse role in the prevention of AD.

Compounds	Class	Mechanism of action	Reference
Ginkgolides	Terpenoid	It enhances the cognitive parameters by enhancing the cholinergic transmission in AD model. Moreover, it substantially diminishing the neurotoxicity, level of proinflammatory cytokines by inhibiting the neuronal MAPK pathway. Further it also decreases the excitotoxic damage caused by glutamate and has antioxidant and antiapoptotic properties	(Kavitha et al., 2020)
Rosmarinic acid	Polyphenol	It decreases the hyperphosphorylation of the tau protein, prevents fibrilization in vitro and reduces β-sheet assembly in tau protein linked to AD	(Alkam et al., 2007)
Resveratrol	Polyphenol	It reduces the aggregation of Aβ and its toxicity and neuroinflammation in brain, thereby substantially play a substantial neuroprotective role in the progression and treatment of moderate to mild AD. It reduces the action mechanism of free radicals and pro-inflammatory proteins by inhibiting the expression of glutathione and COX-2. Moreover, it diminishes the activity and secretion of TNF-alpha and IL-1, thus reducing the pathophysiology of AD.	(Komorowska et al., 2020),(Gu et al., 2021).
Curcumin	Polyphenol	It inhibited the phosphorylation and degradation of IϏB and the nuclear translocation of NF-B p65	(Ahmed et al., 2021)
Docosahexaenoic acids	Polyunsaturated fatty acid	Reduce the oxidative stress and lipid peroxide, also downregulate the β- and γ-secretase activity and enhance the cleaving activity of α-secretase	(Grimm et al., 2016).
Punicalagin	Ellagitannin	Reduces the level of β-secretase, thus decreases the level of β-amyloid plaques.	(Andrade et al., 2019)
Rhodosin	Flavanol	Increases the learning and cognitive behavior mainly due to their antioxidant activities	(Andrade et al., 2019)
Morin	Flavonoid	Inhibit the secretion of β-amyloid plaques in vitro and downregulates Tau protein's hyperphosphorylation in vivo.	(Choudhury et al., 2017)
1,2,3,4,6-Penta-O-galloyl-β-d-glucopyranose	Polyphenol	Inhibit the oligomerisation of Aβ, thus prevent and destabilize the formation of Aβ fibrils. Therefore, reduce the Aβ plaques and enhance the cognitive behaviors.	(Li et al., 2022)
Pterostilbene	Polyphenolic compound	Pterostilbene inhibit the secretion of IL-1β, IL-6, IL-3 and TNF-α, thus reduce the cytotoxicity in cells. Moreover, it also reduces and inactivates the NLRP3/caspase-1 inflammasome.	(Li et al., 2018a)
Salidroside	Glucoside	Salidroside substantially enhance the cognitive behavior in vivo model by regulating the expressions of thioredoxin, thioredoxin associating protein and proteins of NF-ϏB pathway such as p65, NF-ϏB, IKKα, IkB- α, and IKKβ.	(Gao et al., 2015)
Paeoniflorin	Terpene	Paeoniflorin improved memory impairments and lowered A-β accumulation by inhibiting the expression of glycogen synthase kinase-3 (GSK-3), NLRP3 inflammasome, and several cytokines for instance, IL-1β and TNF-α along with the activation of NF-ϏB.	(H.-R.Zhang et al., 2015)
1,8-Cineole	Terpene	It downregulates the mitochondrial membrane dysfunction, NO and ROS levels. It also reduces the expression of several pro-inflammatory cytokines such as IL-1, TNF-α, NF-ϏB and IL-6, COX-2, and NOS-2.	(Khan et al., 2014)
L-Theanine	Amino acid in nature	It substantially diminished the level of A1–42 in brain and also reduce the memory disorders in mice, thus reducing the neuronal death in the hippocampus and cortex regions. In addition, it also dysregulates the expression of some extracellular signal-regulated kinase (ERK), MAPK, p38, and NF-ϏB along with the expression of oxidative biomarkers, glutathione and lipid damage in the brain.	(Kim et al., 2009)

**Fig. 5:**
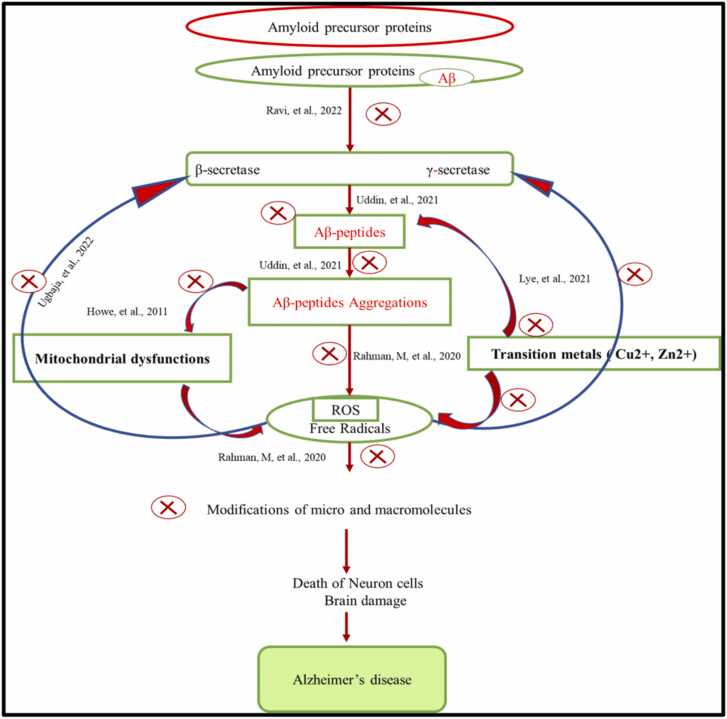
Mechanism of phytochemicals in the prevention of pathophysiology of AD. (The red cross shows the prevention or inhibition process by phytochemicals).

Thus, various epidemiological studies have suggested that consuming lots of vegetables and fruits, which have different categories of bioactive phytochemicals, and these phytoconstituents plays a substantial role in lowering and controlling the onset/risk of AD. Consequently, several studies show the ameliorating the effect of phytochemicals by lowering the pathophysiology of AD by reducing or inhibiting the effect or generation of oxidative stress, mitochondrial dysfunction, damages in DNA, RNA, Proteins, lowering the effect of transition metals and inhibiting the aggregation of β-amyloids and NFT (Fig. 5). Besides, some in vitro and in vivo models have also provided strong witness that plant/phytochemical can lower the neuropathology and pathophysiology of AD.

## 7 Conclusions

Based on genetic, biochemical, and histopathological data, the amyloid cascade theory and formation of neurofibrillary tangles in Alzheimer's disease was suggested 30 years ago. However, multiple groups of anti- Aβ and NFT medicines that influence the development, accumulation, and clearance of Aβ and NFTs have failed in clinical trials for over 15 years. Few drugs that block the development of Aβ, such as *BACE* and γ-secretase inhibitors, have been studied to expedite cognitive failure. Advances in fundamental science and molecular diagnostics have raised tremendous opportunities for the production of medicines. In the context of the G8 declaration, there is no time to waste in seeking to come up with a preferable future for the patients of tomorrow, while ameliorating treatment and support for today's patients should remain a priority. Moreover, some phytochemicals also show significant anti-Alzheimer’s activity by inhibiting the aggregation of beta amyloid and Tau phosphorylation, by reducing the expression of various cytokines and enhance the expression of various enzymes thus, reinstate the generation and function of neurotransmitters. It is clear that oxidative stress, along with Aβ and p-tau toxicity, is a significant factor in the development and progression of AD and eventually death of patients suffering from this despicable neurodegenerative disease. Medicinal plants have shown to be a valuable source of important lead compounds over time, and they could be used to build a more potent medicine that is neither cytotoxic nor genotoxic. However, all efforts should be focused on developing neurodegenerative drugs that are enough permeable to pass the blood-brain barrier while being safe and accessible. Therapies approved by the current Food and Drug Administration lack effectiveness and have adverse side effects thus, requiring new methods to tackle AD pathophysiology. However, more research should be conducted to shed more information on the effect of phytochemicals on neurodegenerative disease.

## Funding

This work did not receive any specific grant from funding agencies in the public, commercial, or profit sectors.
